# Synthesis and Characterization of Magnetic Nanoparticle-Decorated Multiwalled Carbon Nanotubes

**DOI:** 10.1021/acsomega.4c05027

**Published:** 2024-09-16

**Authors:** Lynn Hein, Sylvain Coulombe

**Affiliations:** Catalytic and Plasma Process Engineering, Department of Chemical Engineering, McGill University, Montréal, Québec H3A 0C5, Canada

## Abstract

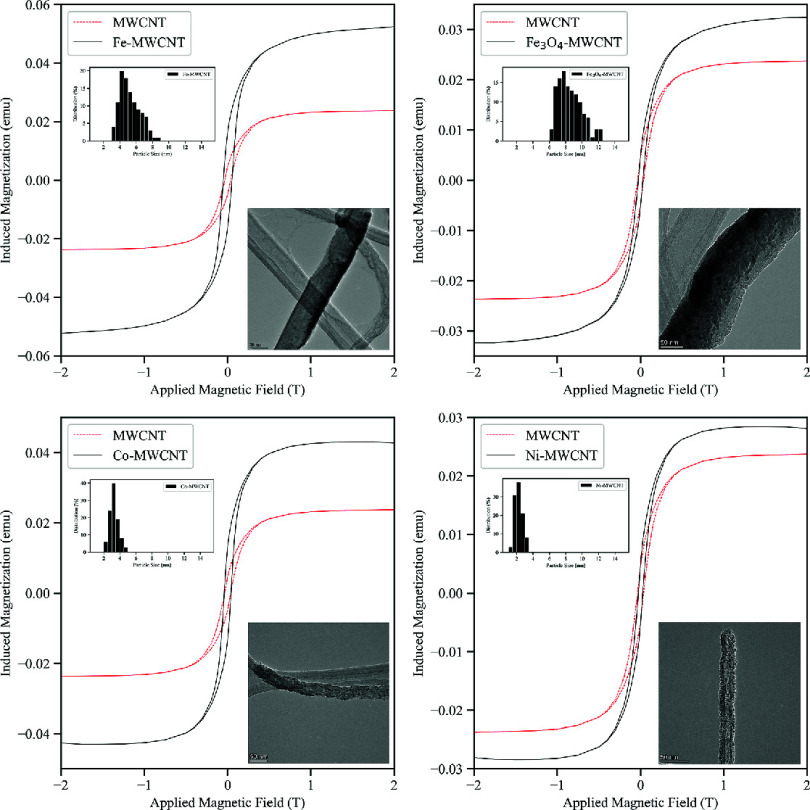

Multiwalled carbon nanotubes find applications in many fields due to their extraordinary properties. However, depending on their synthesis method, they show no or a poor response to the presence of a magnetic field. This limits their usability in magnetic applications. In this study, the maximum induced magnetization of multiwalled carbon nanotubes was increased by deposition of magnetic nanoparticles, which were produced by nanosecond pulsed laser deposition under inert low-pressure conditions using iron (Fe), magnetite (Fe_3_O_4_), cobalt (Co), and nickel (Ni) targets. Extensive chemical and physical characterization of the added nanoparticles was performed. It was found that for the same synthesis conditions, Fe and Fe_3_O_4_ targets resulted in the formation of larger, asymmetrical magnetic Fe nanoparticles with a Fe_3_O_4_ shell (Fe@Fe_3_O_4_) (3.2–8.6 nm) and Fe_3_O_4_ (6.0–12.4 nm) nanoparticles, respectively. Smaller, more spherical Co@CoO (2.1–5.0 nm) and Ni@NiO (1.4–3.5 nm) nanoparticles were obtained from the Co and Ni targets, respectively. The highest increase in maximum induced magnetization was observed for multiwalled carbon nanotubes with Fe@Fe_3_O_4_ (5.37 ± 0.15 emu/g) or Co@CoO nanoparticles (4.29 ± 0.01) compared to pristine multiwalled carbon nanotubes (2.46 ± 0.08 emu/g) and nanotubes with Fe_3_O_4_ (3.79 ± 0.38 emu/g) or Ni@NiO nanoparticles (2.85 ± 0.06 emu/g). Finally, superior adhesion of the Fe@Fe_3_O_4_ and Fe_3_O_4_ nanoparticles to multiwalled carbon nanotubes compared to the Ni@NiO and Co@CoO nanoparticles was identified.

## 1 Introduction

The synthesis of carbon nanotubes (CNTs) was first reported by Iijima in 1991.^1^ Ever since their discovery, CNTs have been further characterized and have found many applications due to their unique property pool. They have received a lot of attention as a support structure for other nanoparticles and molecules due to their extremely large surface area and native inertness. Other noteworthy properties include a high aspect ratio, high mechanical strength, good thermal and electrical conductivities, as well as excellent chemical tuneability.^2^ In recent years, the preparation and potential applications of magnetic CNTs have received considerable attention. In nanomedicine, magnetic CNTs are developed for targeted drug delivery by guiding magnetic CNTs to their treatment destination using externally applied magnetic fields.^3−6^ Due to their unique dimensions and physical properties, magnetic CNTs are also used as catalyst particles, magnetic solid-phase extraction absorbents, sensors, antibacterial agents, and magnetic resonance imaging contrast media.^7^

As-grown CNTs can respond to magnetic fields depending on their synthesis method. Lipert et al. reported that CNTs grown by chemical vapor deposition in the presence of iron (Fe) catalyst particles have ferromagnetic properties. Specifically, single-walled and multiwalled carbon nanotubes exhibited maximum induced magnetic saturation values of 5.93 emu/g and 0.16 emu/g, respectively.^8^ An improvement in the magnetic properties of CNTs generally entails the addition of magnetic nanoparticles (NPs) into or onto individual nanotubes. Most synthesis routes for magnetic NP production follow bottom-up approaches. Specifically, NPs form as the nucleation of supersaturated species is triggered, following a primer reaction or the evaporation of solids.^9,10^ The chemical and physical events leading to NP formation can take place in a gaseous or liquid environment. Magnetic NPs can be produced by solvothermal methods, pulsed laser ablation, chemical vapor deposition, electrochemical processes, and copolymerization or coprecipitation reactions.^11^ Dry (gas phase) methods are preferred as they allow for the production of pure NPs. The NP purity depends on the purity of the bulk source material and the gas phase without risk of contamination from clean reactor walls.^12^ In the synthesis of magnetic CNTs, dry processes also prevent the agglomeration of pristine, hydrophobic CNTs that would occur in the presence of a polar liquid. Nanosecond pulsed laser deposition (PLD) in a low-pressure inert gas environment was employed as the synthesis method in the present investigation. This technique was first tested in 1965 and has become increasingly popular over the years.^13^ Depending on the chamber pressure and laser conditions, thin metallic films to well-dispersed NPs as well as coarse NP deposits can be obtained. Pajootan et al. showed that the morphology of the NPs produced by PLD changes drastically from smooth well-dispersed NPs to coarse NP films by varying the chamber pressure from 10^–5^ to 1 Torr and the deposition time from 1 to 10 min while keeping all other parameters constant.^14,15^ Further benefits of PLD are the absence of solvents, short deposition times and a wide range of target materials that can be used.

In their bulk form, most magnetic materials exhibit ferro- or ferrimagnetism. As a result, they experience strong attraction to a magnet. The bulk phase is composed of many single magnetic domains which have permanent magnetic moments (even when an external magnetic field is not present). The magnetization of ferro- or ferrimagnetic materials is the vector sum of all those magnetic moments. Magnetic particles with a diameter below 1 μm are of the size of a single magnetic domain. As such, they magnetize uniformly across their entire size. If the particle size decreases below a critical value (typically 3–100 nm, depending on the material), they can exhibit superparamagnetism.^16^ This behavior is characterized by the particle’s magnetic moment randomly overcoming the associated energy barrier and changing to the opposite direction. The time it takes for the magnetization direction to flip depends on temperature and the NP properties and is referred to as the Néel relaxation time (*τ*_*N*_). When measuring the magnetization of superparamagnetic NPs at a given temperature, the recorded response depends on the measurement time (*τ*_*M*_). If *τ*_*M*_*≫ τ*_*N*_, the net measured magnetization is around zero in the absence of an external field since the direction of the magnetic moment continuously flips. In this case, the NPs are said to be in their superparamagnetic state. Contrarily, if *τ*_*M*_*≪ τ*_*N*_, the direction of the magnetic moment remains constant throughout the measurement and the NPs are said to be in their blocked state.^17^ Superparamagnetic NPs have similar behavior to paramagnetic materials, with the difference that their saturated magnetization is high and their response to an externally applied magnetic field is sharp, i.e., they retain relatively high magnetic susceptibility.^18^

Previous research has mostly focused on preparing suspended magnetic CNTs by wet chemical processes. In this study, we deposited Fe, magnetite (Fe_3_O_4_), cobalt (Co) and nickel (Ni) -based NPs by PLD on multiwalled carbon nanotubes (MWCNTs). Magnetic NP-covered MWCNTs were prepared to increase the maximum induced magnetization under the application of a magnetic field compared to pristine MWCNTs and are hereinafter referred to as m-MWCNTs. First, the PLD process was characterized. Then, the morphology and elemental composition of the magnetic NPs were analyzed. Extensive material characterization using transmission electron microscopy (TEM), energy-dispersive X-ray spectroscopy (EDS), selected area electron diffraction (SAED), X-ray diffraction (XRD), X-ray photoelectron spectroscopy (XPS), and thermogravimetric analysis with temperature-programmed oxidation (TGA-TPO) was carried out. The maximum magnetization saturation of pristine and m-MWCNTs was assessed with a vibrating sample magnetometer (VSM). Lastly, the adhesion of the Fe, Fe_3_O_4_, Co and Ni-based NPs to individual MWCNTs was examined by sonicating the m-MWCNTs in water and by evaluating the filtered testing solutions using inductively coupled plasma optical emission spectroscopy (ICP-OES) for the presence of the respective metallic elements.

## 2 Materials and Methods

### 2.1 Preparation of m-MWCNTs

MWCNTs were grown directly on 3.0 cm × 4.0 cm 316L stainless steel (SS) mesh coupons (wire diameters: 0.07 and 0.05 mm, grid opening: 0.015 mm, thickness: 0.150 mm, TWP Inc.) using a thermal chemical vapor deposition batch process previously described elsewhere.^15^ The iron islands formed by preheating the SS acted as catalytic sites for MWCNT growth in a carbon-rich atmosphere (Ar/C_2_H_2_ in the present study). This synthesis method gave rise to MWCNTs that were 30–40 nm in diameter and 15–20 μm in length. The as-produced MWCNT-coated 316L SS samples were fixed on a substrate holder and inserted in the PLD chamber. The chamber was flushed with argon (UHP, MEGS Specialty Gases) for 3 min to remove residual air and other possible contaminants. Then, the chamber was pumped down to a base pressure of 10^–5^ Torr using a turbomolecular pump (HiPace 80, Pfeiffer Vacuum). All PLD experiments were performed with a nanosecond pulsed Nd:YAG laser (Quantel, Brilliant B10) and with argon as a background gas at 10^–5^ Torr. The laser wavelength, pulse frequency and pulse duration were 355 nm (3rd harmonic of the Nd:YAG laser), 10 Hz and 5 ns, respectively. A series of lenses and mirrors was used to focus the laser beam at an incidence angle of approximately 45° onto the target. The highest laser energy of 96 mJ per pulse was used. The spot size diameter was measured to be ∼1 mm, resulting in a laser fluence of ∼122 mJ/mm^2^. The target holder was moved vertically using a motorized stage to avoid stationary ablation and thermal effects. The laser target and sample holder were positioned face-to-face, and separated by 3 cm. The sample holder was manually rotated around its axis to ensure uniform NP deposition on the MWCNT-covered 316L SS coupon. Specifically, Fe-MWCNTs, Fe_3_O_4_-MWCNTs, Co-MWCNTs and Ni-MWCNTs were produced using magnetic 99.9+% pure Fe (Kurt J. Lesker), 99.9% pure Fe_3_O_4_ (MSE Supplies), 99.99% pure Co (Kurt J. Lesker) and 99.995% pure Ni (Kurt J. Lesker) targets, respectively. Before use, each target was polished using 600 and 1600-grit papers consecutively and sonicated in acetone for 15 min. The ablation time was set to 25 min (15000 laser shots) for each target. After ablation, the samples were removed from the PLD chamber and temporarily stored in sealed Petri dishes until characterization.

**Figure 1:**
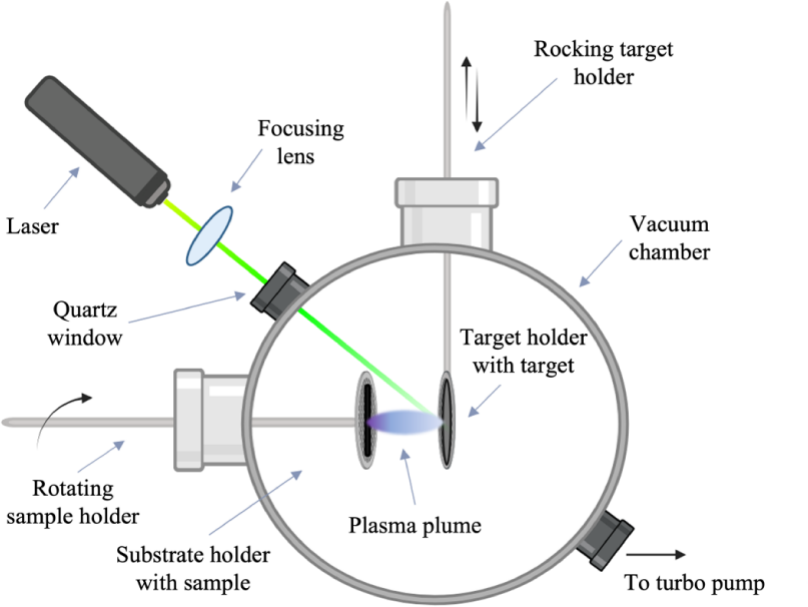
Schematic diagram of the PLD chamber. As the laser hits the target surface, a plasma plume forms and NPs are ejected onto the sample which is facing the target.

The three main steps in PLD start with the interaction of the laser beam with the target. Second, while species are evaporated from the target material and condense as they are projected away from it, a laser-induced plasma plume forms. Lastly, NPs produced in-flight eventually deposit on the substrate facing the target.^19,21^ A schematic representation of a dry PLD setup is shown in Figure 1. By tuning the laser (fluence, wavelength, pulsing frequency, pulse width, spot size) and process (background gas, background pressure, target material, distance of sample to target, sample or target motion) parameters, it is possible to alter the synthesis conditions to obtain individual NPs or thin films of diverse morphologies and properties.^12,20,24^

### 2.2 Characterization of the PLD Process

The average laser powers for different delay times between the flashlamp and the optical Q-switch (Q-switch delay) were measured using a single-channel laser power meter (SOLO model, Gentec-EO). The power meter was zeroed prior to every measurement and the inherent error of the device was ±0.3 mW. Subsequently, the laser energy and peak powers were calculated. Next, the threshold laser fluences for Fe, Fe_3_O_4_, Co and Ni ablation in vacuum were determined experimentally using a high-speed imaging camera (FastCam Mini Ax200, HIS). The deposition rates were determined for every target to accurately predict the resulting masses of NPs on the MWCNTs. To do so, the mass of deposited NPs was confirmed by weighing the MWCNT-covered SS mesh before and after PLD using a microbalance for a range of 750 to 15 000 laser shots. This procedure was carried out for NP deposition from Fe, Fe_3_O_4_, Co and Ni targets. The ablation line depth of every target after 15 000 laser shots was measured using a profilometer (DektakXT, Bruker), as well as the depth measurement tool of an optical microscope (VHX 5000, Keyence). Finally, the composition of the background gas after flushing with argon and before PLD was assessed for the presence of residual atmospheric oxygen and nitrogen. For that, a capacitively coupled radiofrequency plasma was sustained in argon at 20 W and 100 mTorr before PLD and its emission spectrum was captured by optical emission spectroscopy (OES) using a UV–vis spectrometer (Ocean FX-XR1, OceanOptics).

### 2.3 Characterization of m-MWCNTs

The morphology, size distribution, chemical composition and crystalline structure of the deposited NPs were investigated by TEM (Talos F200X G2, Thermo Scientific) at a beam energy of 200 kV coupled with EDS and SAED. For TEM analysis, a few m-MWCNTs were detached from each SS mesh coupon by sonication in acetone for 5 s. A 300 mesh TEM copper grid (Ted Pella Inc.) was immersed in the suspension to collect individual NP-covered MWCNTs. For all other characterization techniques, the m-MWCNTs were analyzed as dry powders. To retrieve m-MWCNT powders, as-grown MWCNTs were first removed from the SS mesh by sonication in solution for 60 min during which the MWCNTs broke off the SS mesh along any point of their length. After sonication, the MWCNTs were removed from the suspension by filtration using 0.45 μm filters (Cytiva Whatman, Fisher Scientific). The filters with the MWCNTs were then inserted in the PLD chamber for NP deposition. Finally, the m-MWCNTs were removed from the filter as dry powders. As such, interference with the SS substrate during analysis and misinterpretation of the characterization results were avoided. XRD (D2 Phaser, Bruker) was performed using a Cu X-ray source and monochromatic X-ray optic to confirm the crystalline structure of the deposited NPs. The background of each diffraction pattern was removed manually and peak deconvolution was performed. The XRD patterns were compared to reference patterns from the Crystallography Open Database (COD). In addition, the oxidation states of the NPs were studied by XPS (K-Alpha XPS apparatus, Thermo-Scientific) with an Al Kα source, microfocused monochromator, and spot size of 200 μm. To avoid charging effects during data acquisition, the flood gun was turned on. The analysis of all spectra was carried out using the Avantage software. To further investigate the iron oxide phases present in the NPs originating from the Fe and Fe_3_O_4_ targets, TGA-TPO (TG 209 F1 Libra, Netzsch) was performed. Measurements were carried out in air from room temperature to 900 °C with a heating rate of 10 °C/min. The magnetic properties of pristine and m-MWCNTs were evaluated using a VSM (Model EV9, ADE technologies) with magnetic field strengths up to 2 T and a frequency of 75 Hz at room temperature. Lastly, the adhesion of the deposited NPs to the MWCNTs was assessed by sonicating m-MWCNTs in reverse osmosis water for 60 min. The filtered testing solutions were digested in concentrated nitric acid and evaluated for traces of Fe, Co, and Ni by ICP-OES.

## 3 Results and Discussion

### 3.1 PLD Process

The average laser powers *P*_*a*_ [W] for different Q-switch delays were measured. The laser energies per pulse *I* [J/pulse] and peak laser powers *P*_*max*_ [W] were calculated with eqs 1 and 2, respectively. To do so, the pulse frequency *f* [Hz] and the pulse width τ [s] were used. Finally, the laser fluence *F* [J/m^2^] was determined by dividing the laser energy per pulse by the spot size area *A* [m^2^/pulse] as described by eq 3.^25^
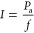
1
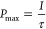
2
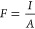
3

**Figure 2:**
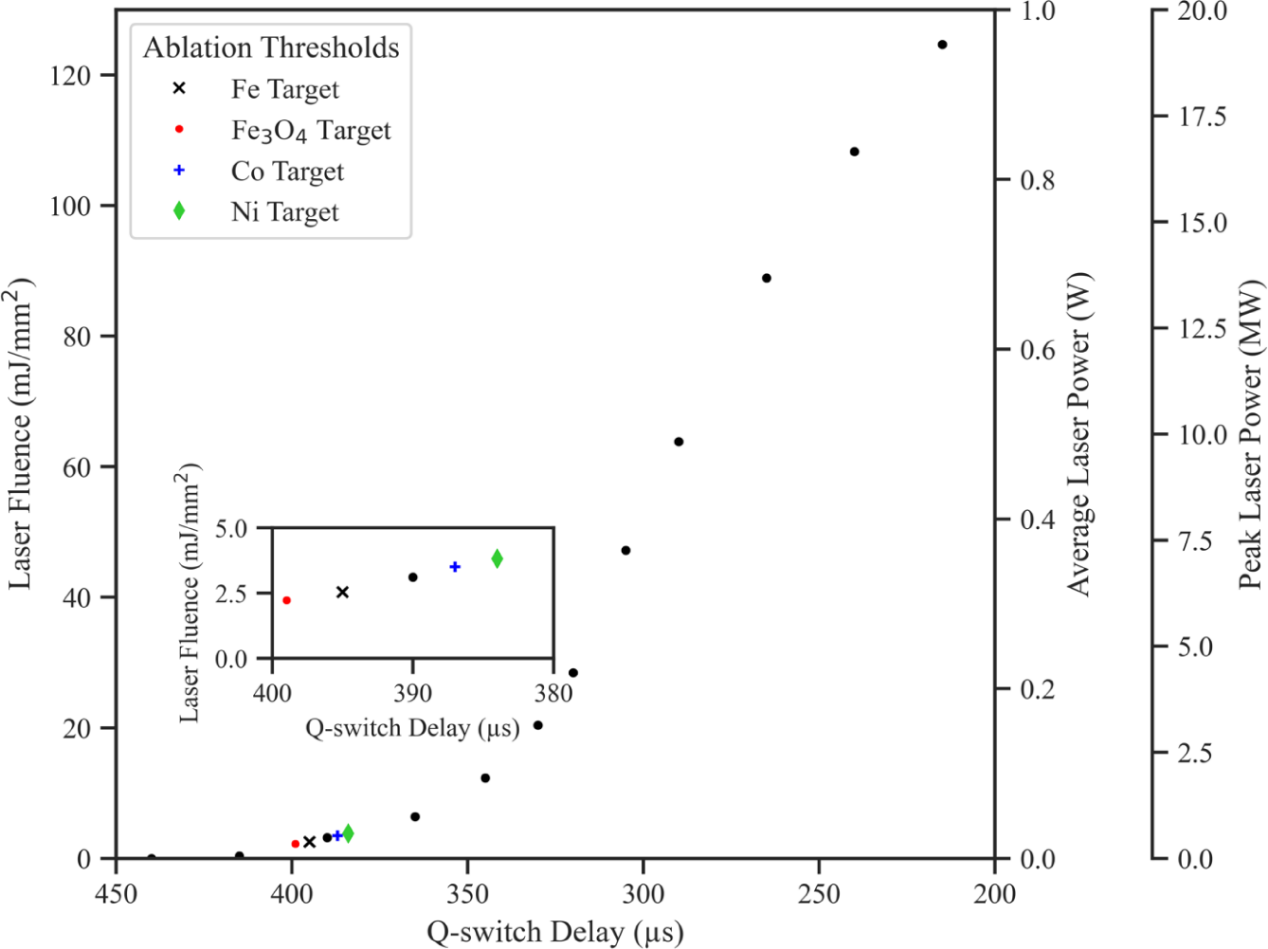
Effect of the Q-switch delay time on the laser fluence and peak laser power with threshold ablation fluences for the Fe, Fe_3_O_4_, Co, and Ni targets. The laser fluence and peak laser power increase nonlinearly with a decreasing Q-switch delay time.

The measured and calculated values are displayed in Figure 2. It becomes apparent that the average power was negligible for Q-switch delays above 400 μs and increased nonlinearly for shorter delays. The shortest Q-switch delay of 215 μs produced a laser pulse with a fluence of 122 mJ/mm^2^ and a peak power of 19.2 MW. The threshold laser fluence marks the minimum laser fluence required for material ablation from a solid target. The Ni target had the highest ablation threshold laser fluence (3.81 mJ/mm^2^), followed by the Co (3.50 mJ/mm^2^,) Fe (2.53 mJ/mm^2^) and Fe_3_O_4_ (2.22 mJ/mm^2^) targets. The threshold laser fluences and respective Q-switch delays for Fe, Fe_3_O_4_, Co, and Ni target ablation in vacuum are indicated in Figure 2.

**Figure 3:**
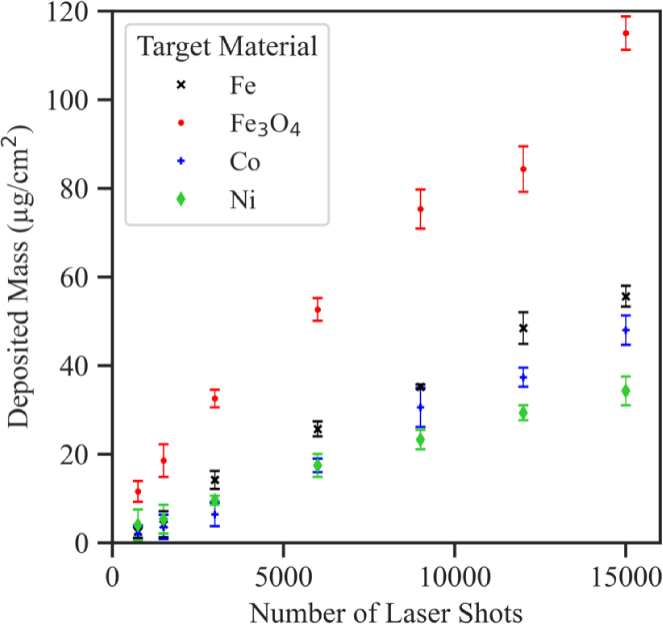
Deposited mass based on the number of laser shots for Fe, Fe_3_O_4_, Co, and Ni targets with all other PLD parameters constant. The respective mass of deposited NPs increases linearly with the number of laser shots and depends on the properties of the target material.

To accurately estimate the mass of magnetic NPs deposited on the MWCNTs for a specific number of laser shots, the total deposited NP mass on the projected MWCNT surface area vs the number of laser shots was plotted and compared in Figure 3. The Fe_3_O_4_ target is accompanied by the highest material deposition rate for identical PLD conditions. While a total NP mass of 115.0 ± 3.8 μg/cm^2^ was deposited on the MWCNT substrate with the Fe_3_O_4_ target, less deposition (in mass) was observed with the Fe (55.6 ± 2.3 μg/cm^2^), Co (48.0 ± 3.3 μg/cm^2^), and Ni (34.3 ± 3.2 μg/cm^2^) targets for 15 000 laser shots.

The deposited NP mass is linearly proportional to the number of laser shots. However, it has been shown previously that at high laser fluences, the deposition yield increases almost exponentially due to nonequilibrium splashing as a result of transient melting.^23^ From Figure 3 it is apparent that the deposition rates for the different targets at a laser fluence of 122 mJ/mm^2^ did not enter the nonlinear regime. The deposition rates were determined by a linear fit of the experimental data. The Fe_3_O_4_ target deposition rate per pulse of 6.89 ng/(cm^2^ pulse) was significantly larger compared to that of the other targets. The deposition rates per pulse for the Fe, Co, and Ni targets were 3.82, 3.31, and 2.27 ng/(cm^2^ pulse), respectively.

Some theoretical parameters can help to better understand the specific trends associated with each target. The laser absorption depth per pulse *Z* [m] is given by eq 4.
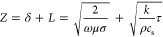
4

Where δ [m] is the skin depth, *L* [m] is the thermal diffusion length, ω is equal to 2π*f*, *f* [Hz] is the radiation frequency, μ [H/m] is the magnetic permeability, σ [1/(Ω·m)] is the electrical conductivity, *k* [W/(m·K)] is the thermal conductivity, ρ [kg/m^3^] is the density, *c*_*s*_ [J/(kg·K)] is the specific heat, and τ [s] is the pulse width. The skin depth typically has a value of approximately 25 nm and can be considered as negligible.^24^ The theoretical irradiated mass per pulse *m* [kg] can then be estimated based on the target density, the spot size *A* [m^2^] and the absorption depth with eq 5.
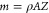
5

Table 1 summarizes the experimental and theoretically calculated data for each target. The references for physical property data used in the theoretical calculations are included in the table for each value.

**Table 1:** Mass Deposition Rate, Ablation Line Depth, Laser Absorption Depth and Irradiated Mass for Fe, Fe_3_O_4_, Co, and Ni Targets

Target	Mass deposition rate per pulse [ng/cm^2^]	Ablation line depth after 15 000 shots [μm]	Laser absorption depth Z per pulse [μm]	Irradiated mass per pulse [ng]
Fe	3.82	25 ± 1.5	13.0^26,28^	80.0
Fe_3_O_4_	6.89	285 ± 13	237.3^26,29^	964.3
Co	3.31	23 ± 2.7	13.6^26,27^	93.8
Ni	2.17	24 ± 0.9	12.6^26,28^	88.0

As mentioned previously, the highest mass deposition was achieved with the Fe_3_O_4_ target for the same number of laser shots. Similarly, the measured ablation line depth after 15 000 laser shots on the Fe_3_O_4_ target was approximately ten times larger compared to the ablation line depths of the Fe, Co, and Ni targets for the same amount of laser shots. These experimental values align with the calculated theoretical values which indicate that the laser absorption depth and the irradiated mass per laser pulse of the Fe_3_O_4_ target are significantly larger compared to the values of the other targets. These differences are due to the physical properties of Fe_3_O_4_ used in eqs 4 and 5 compared to the ones of the metallic targets. One contradiction that may stand out is the inverse relationship of the mass deposition rate and the irradiated mass per pulse for the Fe, Co, and Ni targets. While a higher deposition rate was measured for the Fe target, the Co and Ni targets experienced higher calculated irradiated masses per pulse. This can be explained by the different NP formation mechanisms, as well as NP morphologies and sizes that were ejected from the different targets upon laser irradiation which will be addressed in the next section. It should be noted that the physical process of NP synthesis and deposition by PLD is very complex. The comparison of the experimental against theoretical data is limited and should be regarded as a simplification.

**Figure 4:**
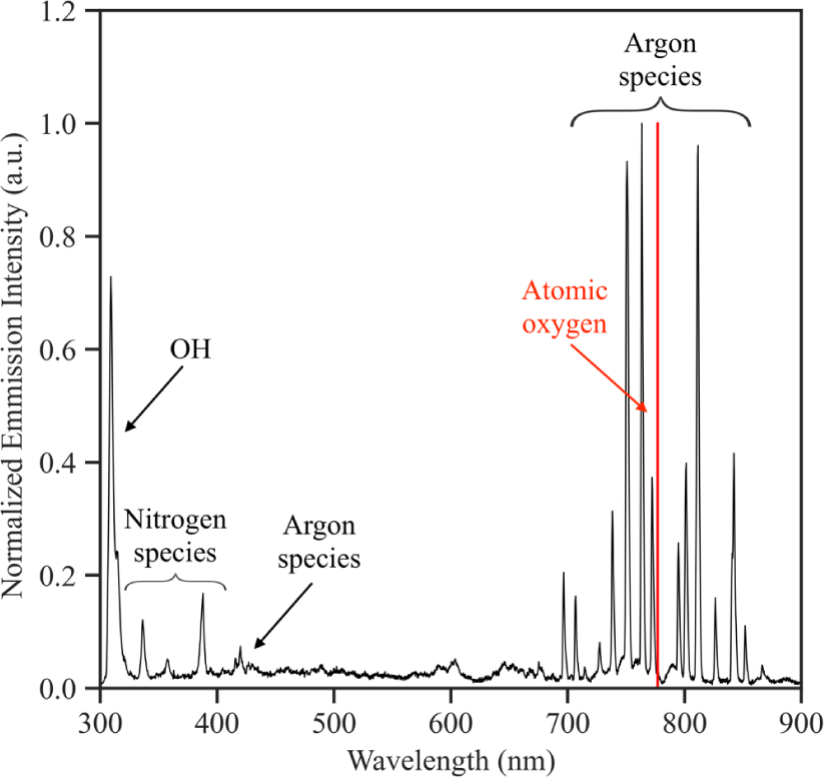
OES spectrum of the PLD chamber background gas. Residual moisture and nitrogen from the atmosphere are still present in the reactor, along with argon which constitutes the background gas. Atomic oxygen which could lead to NP oxidation in the PLD chamber is not detected.

The normalized OES spectrum of the background gas after flushing with argon, after pumping down to 100 mTorr and before PLD is shown in Figure 4. The peaks in the 700–900 nm region and around 425 nm are characteristic of argon which was used to flush the reactor and ignite the plasma. The prominent peak at 310 nm can be attributed to OH species which originate from moisture in the PLD chamber.^30^ Finally, the peaks between 330 and 400 nm are typical of excited, molecular nitrogen.^31^ It is important to note that no atomic oxygen is present in the spectrum which would have had a characteristic main peak at a wavelength of 777 nm, indicated by the red line in Figure 4.^32^ We infer that despite the presence of moisture in the background gas, water was not significantly dissociated into its atomic components during the formation of the laser plasma plume. We also infer that the residual oxygen from air was mostly evacuated from the reactor prior to PLD and that not enough oxygen was present in the PLD chamber to oxidize the newly formed NPs.

### 3.2 m-MWCNT Characterization

**Figure 5:**
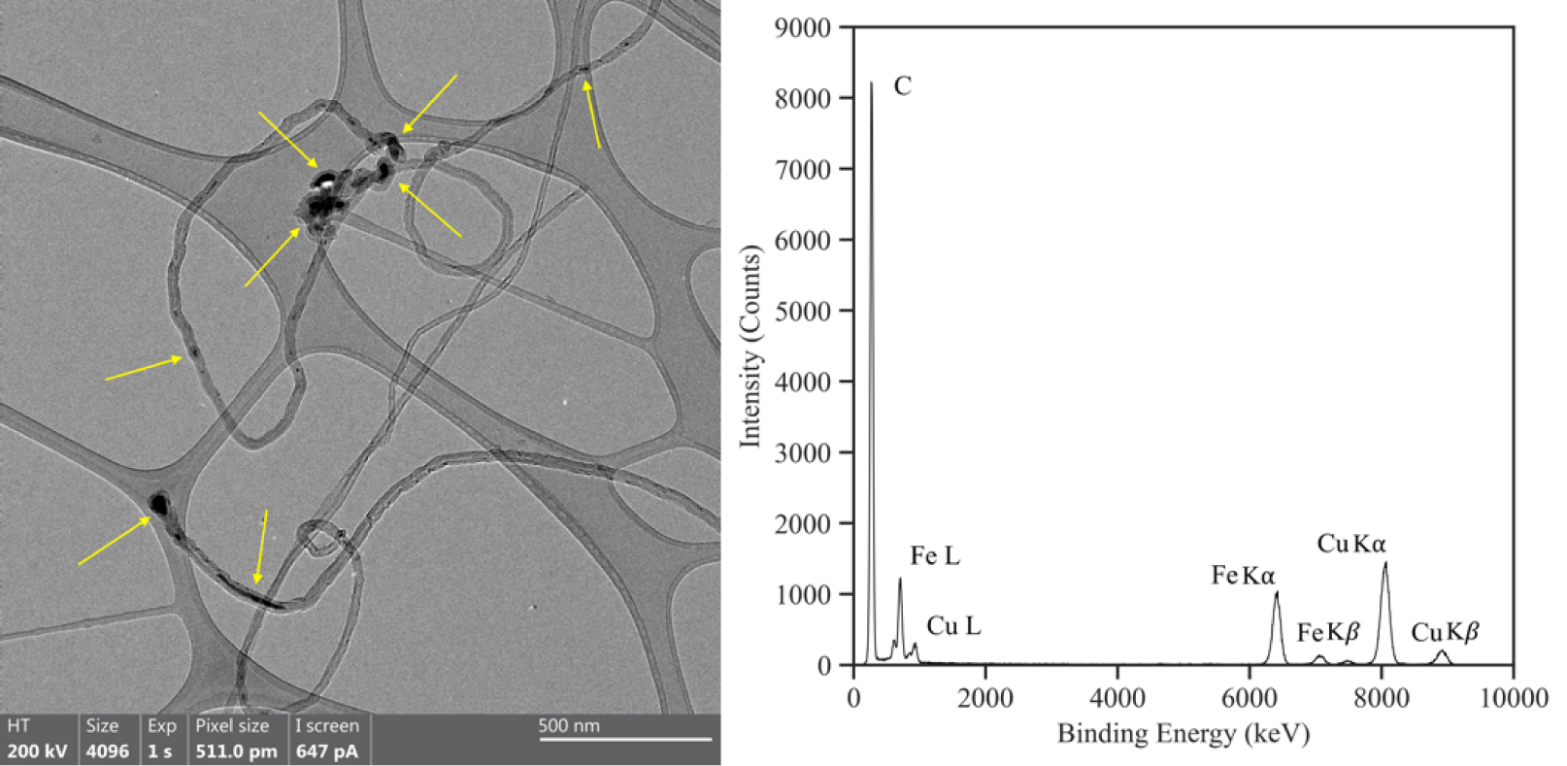
TEM image and corresponding EDS spectrum of as-grown MWCNTs. Fe catalyst particles are trapped inside individual MWCNTs and are free of oxygen.

**Figure 6:**
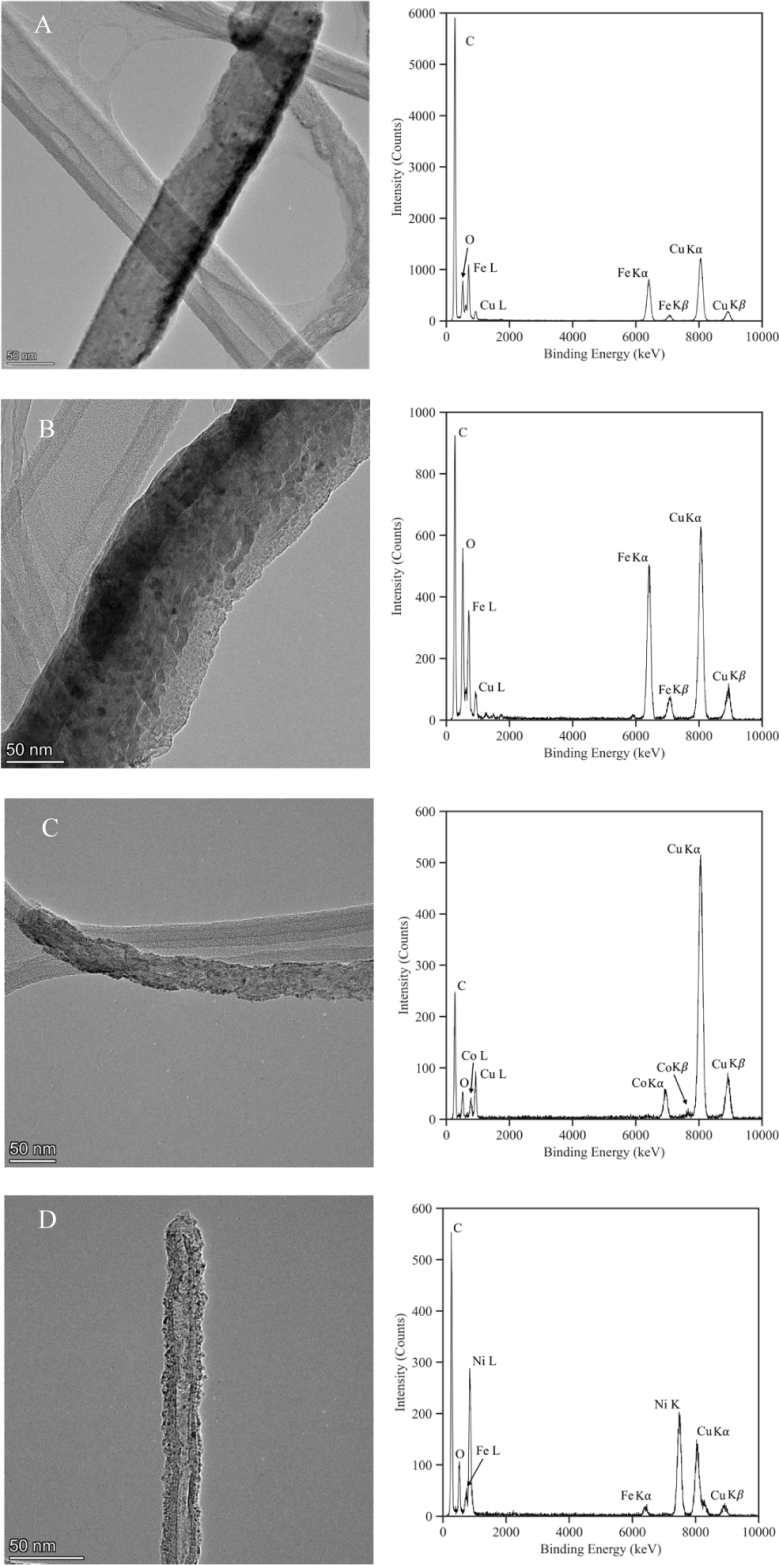
TEM image and corresponding EDS spectrum of (A) Fe, (B) Fe_3_O_4_, (C) Co, and (D) Ni-based NPs on individual MWCNTs. NPs are visible on the outside walls of individual MWCNTs and the presence of oxygen indicates NP oxidation.

First, pristine MWCNTs were analyzed by TEM and EDS. Trapped, pure Fe NPs were visible inside individual MWCNTs as shown in the TEM image in Figure 5. These Fe NPs are catalyst particles and originate from the SS substrate that the MWCNTs were initially synthesized on.^33,34^ The EDS spectrum of pristine MWCNTs prior to the deposition of NPs did not show any oxygen (cf. EDS spectrum in Figure 5). Apart from carbon (from the MWCNTs) and copper (from stray signal of the TEM grid), the as-grown MWCNTs only contained Fe. The same analyses were repeated for the m-MWCNT samples and revealed the different NP morphologies and elemental compositions as displayed in Figure 6. The deposition of NPs from the Fe and Fe_3_O_4_ targets formed a near-continuous film along the MWCNTs, whereas individual NPs originating from the Co and Ni targets were visible on the corresponding samples. For all m-MWCNT samples, the NPs were composed of the elements from the respective targets and the Fe catalyst NPs. In addition, oxygen appeared in the spectrum of all the m-MWCNT samples which indicates oxidation of the NPs added by PLD.

**Figure 7:**
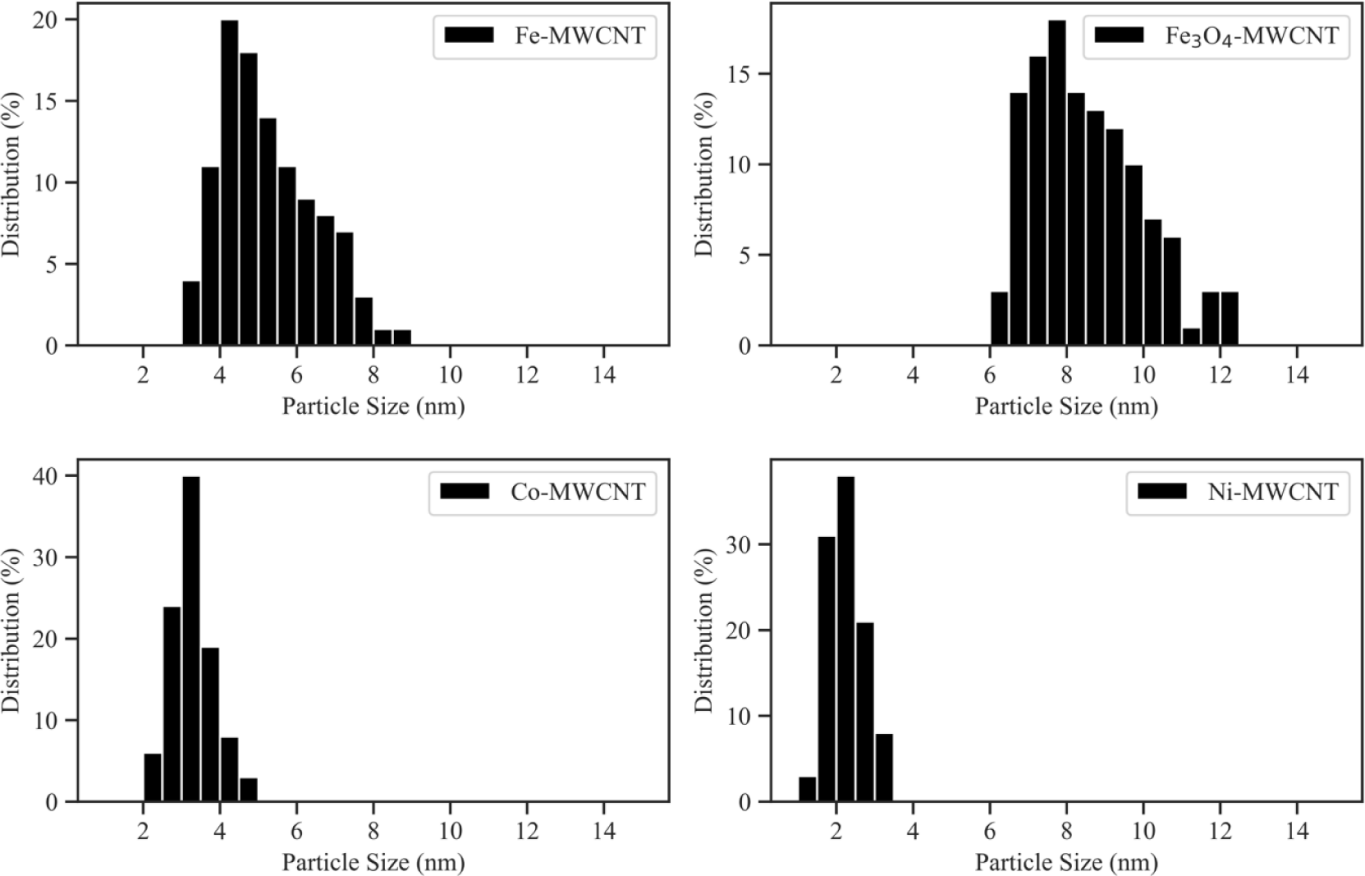
Particle size histograms for NPs from Fe, Fe_3_O_4_, Co, and Ni targets. Larger NPs are accompanied by a wider particle size distribution.

Visually, it was noticeable that the NPs of the Fe-MWCNTs and Fe_3_O_4_-MWCNTs were larger than the ones deposited on the Co-MWCNTs and Ni-MWCNTs. It is also evident that the NPs of the Fe-MWCNTs and Fe_3_O_4_-MWCNTs seemed to have merged with neighboring NPs to form a fused NP layer. To highlight the NP size differences, the average NP sizes and distributions are listed in Table 2, and the matching particle size histograms are displayed in Figure 7. It should be noted that only distinct NPs on the Fe-MWCNT and Fe_3_O_4_-MWCNT samples were considered for particle size measurements. The former are assumed to be representative of the initial particle size of NPs before coalescence with nearby NPs. When comparing the average particle size data, it is clear that the NPs from the Fe_3_O_4_ target were significantly larger compared to all other NPs. They also had the largest particle size distribution. The NPs from the Ni target were the smallest and had the narrowest particle size distribution. The same trend of particle size and particle size distribution was observed for the Fe and Co-based NPs. It has been demonstrated previously that lowering the laser fluence only slightly reduces the average particle size but increases the particle size distribution.^34^ Although the same laser fluence was used for the preparation of every m-MWCNT sample, the particle size distribution varied quite significantly. Since all other PLD process parameters were kept the same, the size differences are attributable to the material properties of the respective targets and the NP formation mechanisms, which will be discussed in the following paragraphs. The particle size histograms were positively skewed for Fe-MWCNTs and Fe_3_O_4_-MWCNTs and nearly Gaussian for Co-MWCNTs and Ni-MWCNTs. Despite the different particle sizes and particle size distributions, the m-MWCNTs were free of μm-sized particles, ejected from the target as droplets which can form at very high laser fluences.^23^ In addition, target surface defects and contamination can contribute to the formation of μm-sized particles. Therefore, it is crucial to pretreat and clean the target surface prior to PLD accordingly.^35^

**Table 2:** NP Size Dimensions

Sample	Average Particle Size (nm)	Particle Size Distribution (nm)
Fe-MWCNT	5.3 ± 1.3	3.2–8.6
Fe_3_O_4_-MWCNT	8.6 ± 1.5	6.0–12.4
Co-MWCNT	3.3 ± 0.5	2.1–5.0
Ni-MWCNT	2.2 ± 0.5	1.4–3.5

To better understand the different NP mean sizes and size distributions, the laser-target interactions need to be considered. These involve thermal events such as material heating, nonequilibrium phase changes, rapid nucleation and superheated liquid generation. When using nanosecond PLD, NPs can be formed as a result of normal surface boiling and evaporation by supersaturation, or phase explosion.^36^ NP formation by supersaturation occurs as the absorbed laser pulse energy first locally heats the target surface to the melting temperature, before eventually reaching the vaporization point. It should be noted that significantly more energy is required to vaporize a metal than to melt it. Most of the laser energy is dissipated via heat conduction into the target material. The amount of energy that is supplied by the laser and effectively absorbed by the target is a determining factor in the formation of NPs. Once the absorbed energy at a given laser fluence exceeds the latent heat of evaporation in vacuum, metal evaporation occurs. If the laser fluence is increased, NPs can be generated by phase explosion.^37^ For the latter mechanism to take place, the temperature of the superheated target material needs to reach a thermodynamic critical point at which it transforms into a liquid/vapor mixture. The homogeneous nucleation rate is then high enough to produce a large quantity of nuclei during very short time intervals. In addition, this process is accompanied by a time delay, called the time lag of nucleation, during which a vapor nucleus grows to a critical size before ejection from the target surface. The resulting NPs have greater size than the nuclei formed during the supersaturation mechanism.^36^ The supersaturation and phase explosion mechanisms will be described in more detail and correlated to Fe, Fe_3_O_4_, Co, and Ni-based NP formation hereunder.

As a high-energy laser pulse hits a light-absorbing metallic target, rapid localized heating takes place leading to sudden material vaporization, and laser-induced plasma plume formation. The plasma plume originates from the collision of expelled target species and surrounding gas molecules. These collisions lead to the excitation of the background gas molecules, initiating cascade generation events of free electrons and ions. The dynamic process of species excitation and deexcitation is accompanied by the emission of photons. The target material, ambient gas composition and pressure, as well as the laser conditions govern the characteristics of the plasma emission spectrum.^12^ The laser plume expansion and confinement are closely related to the formation of two shockwaves: one propagating inside and one propagating outside the target. As the laser hits the target, the laser energy is absorbed which is followed immediately by an energy relaxation event, resulting in the appearance of shockwaves. The plasma plume formation is associated with the propagation of these shockwaves. The expansion velocity of the plasma plume is at an order of magnitude of 10^4^ m/s and its shape is elliptical extending away from the target toward the substrate. This is due to a higher pressure gradient which pulls the plume in the direction perpendicular to the surface.^20,23^ While shorter pulse duration, femto- and picosecond lasers do not interact with the plasma plume, nanosecond lasers experience energy losses through radiation absorption by the plasma plume. As such, thermal energy is generated in the plasma plume and the species number density, as well as the plasma temperature increase. At this time, favorable conditions for NP formation arise. The first mechanism for NP nucleation and growth considered in this work is controlled by energy and supersaturation as described by eqs 6 and 7.
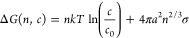
6

Where Δ*G* is the Gibbs free energy [J], *n* is the number of plume species, *k* is the Boltzmann constant [J/K], *T* [K] is the temperature, *c* [mol/m^3^] is the concentration of species, *c*_0_ [mol/m^3^] is the equilibrium concentration, *a* [m] is their effective radius, and σ [N/m] the surface tension.^19^
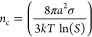
7

S indicates supersaturation and the condition S > 1 must be fulfilled to overcome the nucleation barrier and for the critical particle size *n*_*c*_ to be reached. During supersaturation, nuclei can grow into NPs until the plasma plume extinguishes, the number density decreases, and the interspecies collisions cease. The contributions of electron and ion species should not be underestimated in the nucleation process. As such, the newly formed NPs may reach the MWCNTs as electrically charged NPs.^12^

Co and Ni-based NPs (cf. Figure 6C,D) were smaller and more spherical compared to NPs from the Fe and Fe_3_O_4_ targets. This particle morphology is characteristic of NPs formed by supersaturation. As such, Ni and Co-based NPs were likely formed when the saturation conditions were reached at the Ni and Co target surfaces. Atomic clusters which were ablated from the target surface underwent homogeneous condensation until a critical size was reached. They then behaved as nuclei and grew to a few nm by heterogeneous condensation. As the ejected Ni and Co-based NPs traveled away from the target and to the front of the plasma plume, the effect of the background gas became increasingly more significant. At this stage, condensed NPs grew into larger NPs until they solidified or reached the MWCNTs.^15,21^

Larger asymmetrical NPs as observed in Figure 6A,B for Fe-MWCNTs and Fe3O4-MWCNTs support the assumption of a phase explosion mechanism on the Fe and Fe_3_O_4_ target surfaces. A phase explosion mechanism is more likely for the Fe and Fe_3_O_4_ targets due to lower threshold fluences. Less energy is required to overcome the energy barrier required for material ablation and the energy surplus exceeds the conditions for vaporization by supersaturation. The superheated target surface exceeds the thermodynamic critical point and NP formation by phase explosion takes place. This mechanism is driven by homogeneous bubble formation on the target surface which relaxes abruptly into vapor and liquid nanoclusters. These nanoclusters are quenched from very high temperatures (>5000 K) in just a few microseconds along the perimeter of the plasma plume.^12^ The ejected species can be thought of as material splashes and are already several nm in size when leaving the target surface. This lower threshold fluences and resulting NP formation mechanism also explain why the mass deposition rates of the Fe target were higher compared to one of the Co and Ni targets. Additionally, due to the higher mass deposition rates associated with both the Fe and Fe_3_O_4_ targets, fresh NPs reached the sample surface over previously deposited NPs after a dispersed initial NP layer had formed. The NPs may not have been fully solidified when hitting the MWCNTs. As a result, they disrupted and integrated into the underlying MWCNT structure. Similarly, they fused together with previously deposited NPs which resulted in the formation of a continuous film.

Although the laser energy was kept constant for the different targets, the effective energy absorbed by each target varied which led to different NP formation mechanisms. Static ablation by nanosecond pulses over longer periods of time can develop a significant layer of melted material.^22,23^ To avoid this effect, the target holder was rocked up and down to hinder the development of large heat-affected zones and with that, the extent of localized melting and liquid displacement. The production of finer Fe and Fe_3_O_4_-based NPs by supersaturation could be achieved by lowering the laser fluence. Alternatively, the targets could be moved vertically at a higher pace to overcome the time lag of nucleation. The evaporation rate of the target and NP formation mechanisms further depend on the laser parameters, the properties of the irradiated material, as well as the background gas composition and pressure.^12,23^ These factors should also be considered when attempting to produce NPs by a supersaturation or phase explosion using PLD.

**Figure 8:**
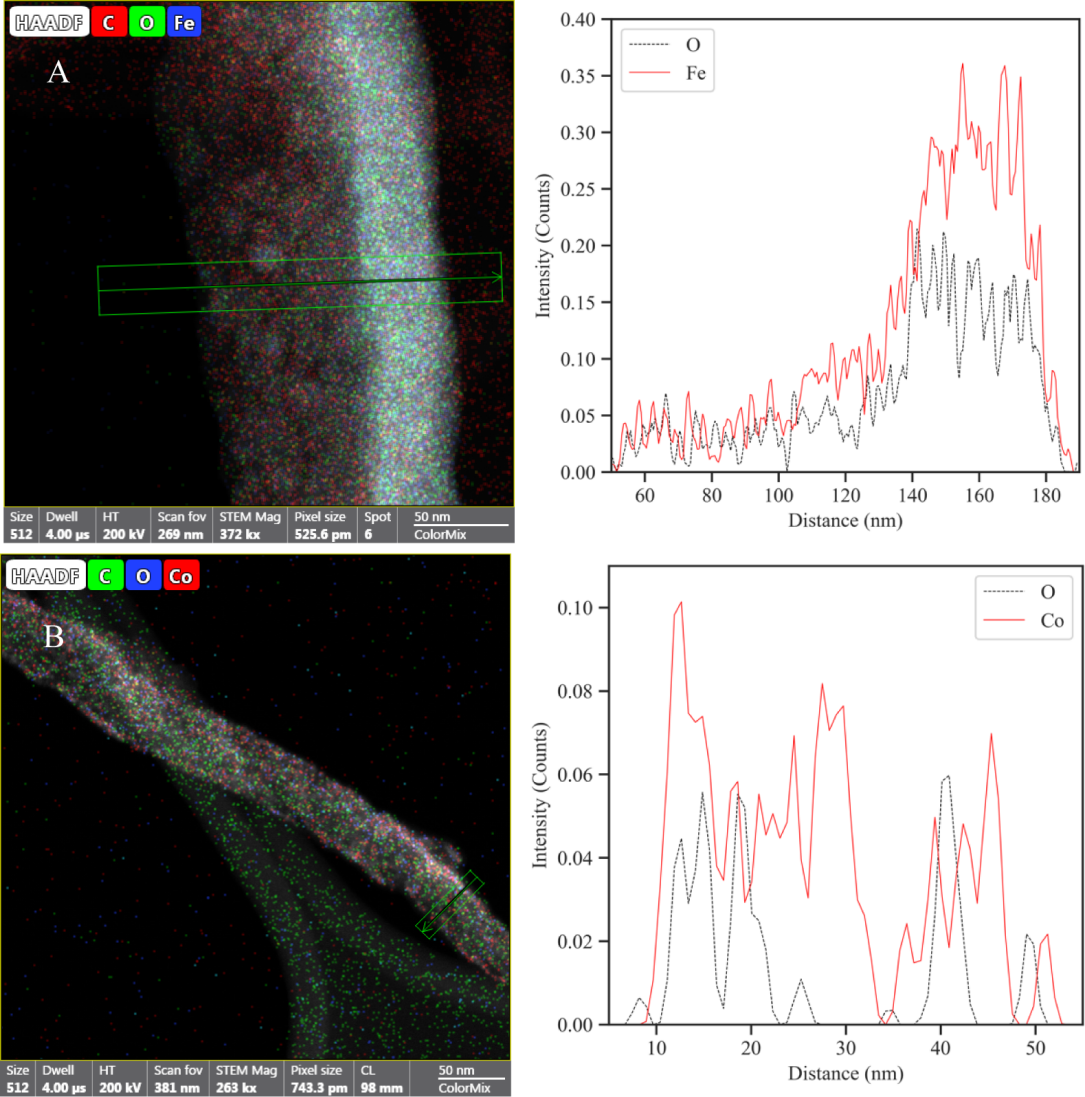
EDS map and line spectrum of (A) Fe_3_O_4_-MWCNTs, and (B) Co-MWCNTs. The NPs from the Fe_3_O_4_ target majorly accumulate along on side of individual nanotubes, while NPs from the Co target spread more uniformly across individual MWCNTs.

The EDS line spectra of m-MWCNTs in Figure 8 confirmed that NPs on the Fe_3_O_4_-MWCNT sample formed a thick layer along the sides of individual nanotubes. This was also observed for Fe-MWCNTs. On the contrary, NPs were well dispersed on individual tubes for Co-MWCNTs. The same deposition pattern was seen with Ni-MWCNTs. The different NP patterns further showcase the two distinct NP formation and deposition mechanisms that were highlighted earlier. For all samples, the presence of oxygen which roughly matched the trend of the metallic elements in the respective line spectra further confirmed the hypothesis that oxidation of the deposited NPs took place.

**Figure 9:**
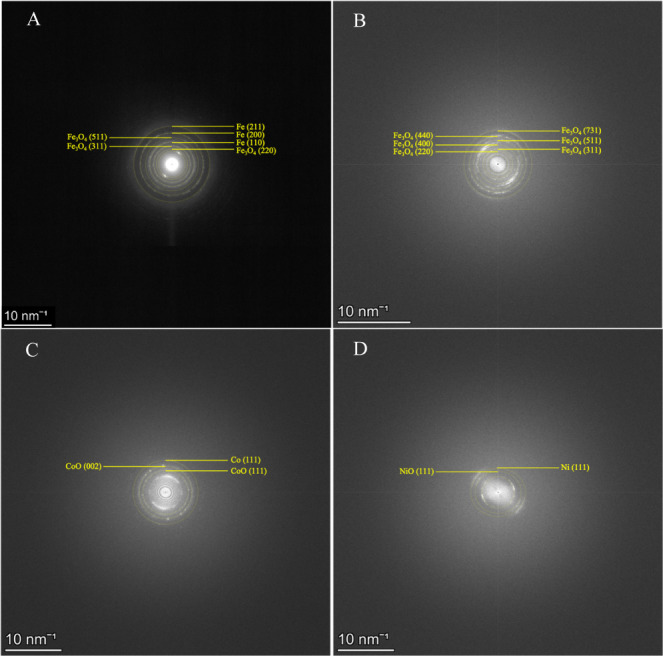
SEAD patterns of (A) Fe-MWCNTs, (B) Fe_3_O_4_-MWCNTs, (C) Co-MWCNTs, and (D) Ni-MWCNTs. Characteristic planes of pure and oxidized metal are identified for NPs from the Fe, Co, and Ni targets. Characteristic planes of the Fe_3_O_4_ phase are detected for NPs from the Fe_3_O_4_ target.

Next, the crystal structures of the deposited NPs were examined. The SAED patterns with the corresponding crystal planes are displayed in Figure 9. The patterns of all m-MWCNT samples were composed of rings which signal the presence of polycrystalline structures. Spotty rings are characteristic of larger, fused grains and were mostly observed in the SEAD pattern of Fe_3_O_4_-MWCNTs.^38^ The radii of the rings were measured to determine the lattice spacings and consequently, the crystal planes of the polycrystalline NPs. A lattice spacing of 0.34 nm was measured in all four SAED patterns and can be correlated to the interlayer spacing of MWCNTs.^39,40^ The NPs originating from the Fe target deposited as Fe@Fe_3_O_4_ NPs (core@shell). NP formation from Fe targets by PLD under low-pressure conditions has been explored far less than in liquid media. PLD in an oxidizing background gas has mostly led to the formation of nonmagnetic hematite (α-Fe_2_O_3_) NPs due to the instantaneous oxidation of newly formed NPs.^41^ Due to the absence of atomic oxygen in the PLD chamber, we deduce that the NPs deposited as pure Fe NPs on MWCNTs and oxidized afterward. At high laser fluences, the plasma plume temperature and shape created appropriate conditions for pure Fe NP formation.^35^ Upon repressurizing the chamber with ambient air, the NP surfaces formed an oxide layer which was majorly composed of Fe_3_O_4_, with the elemental composition of the NP core remaining unchanged. The NPs from the Fe_3_O_4_ target deposited as Fe_3_O_4_ NPs and did not further oxidize into oxygen-richer iron oxides. Finally, the NPs produced from the Co target deposited as Co@CoO NPs, and the ones from the Ni target as Ni@NiO NPs. Similar to the NPs of the Fe-MWCNT sample, the Co and Ni-based NPs first deposited as pure metal NPs inside the PLD chamber. Upon contact with ambient air, they oxidized to form Co@CoO and Ni@NiO NPs. Kim et al. previously reported the synthesis of Ni NPs with a NiO shell by PLD due to surface oxidation.^12^

**Figure 10:**
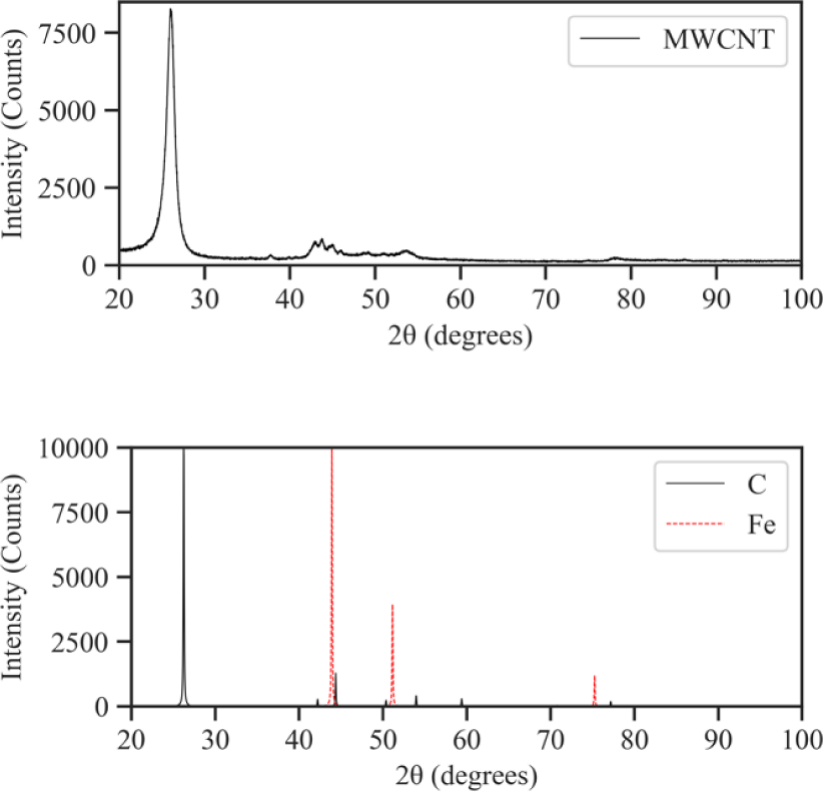
Diffraction pattern of MWCNTs. Overlap exists between the MWCNT diffraction pattern and reference C and Fe diffraction patterns due to the presence of graphitic carbon and Fe catalyst NPs.

**Figure 11:**
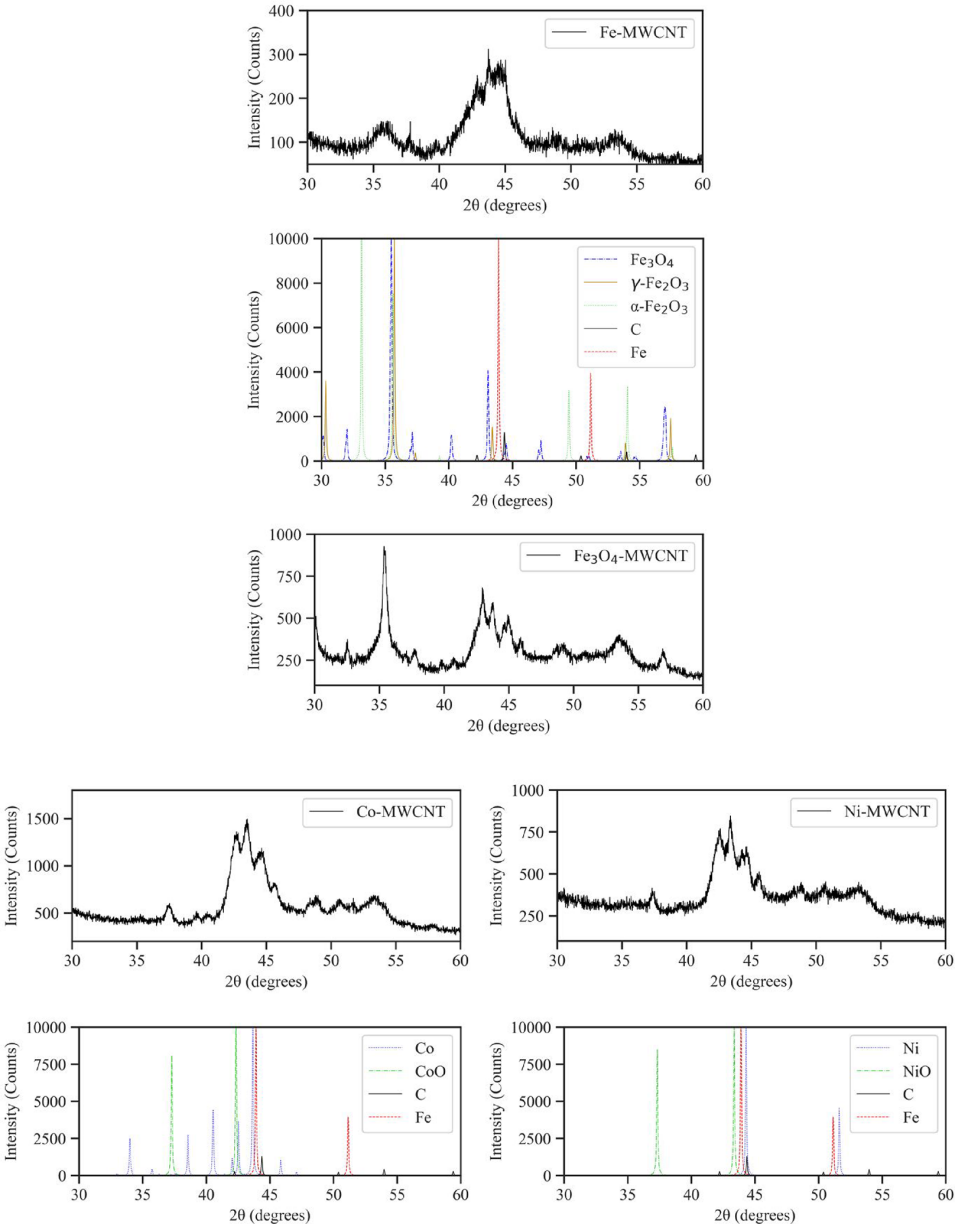
Diffraction patterns of Fe-MWCNTs, Fe_3_O_4_-MWCNTs, Co-MWCNTs, and Ni-MWCNTs. The presence of the deposited NPs is confirmed when comparing the Fe-MWCNTs, Fe_3_O_4_-MWCNTs, Co-MWCNTs, and Ni-MWCNTs diffraction patterns with the respective reference patterns.

Figure 10 displays the XRD diffraction pattern of pristine MWCNTs, as well as the reference patterns of graphite (COD 1 200 017) and pure Fe (COD 9 016 291). The prominent peak at 26.2° was attributable to the graphitic structure of the MWCNTs. The wide peak which spanned from 41.9° to 46.2° appeared due to signal scattering from the MWCNTs and Fe catalyst NPs. This broad peak contained the main Fe peak at 43.7°, as well as additional graphite peaks at 42.2° and 44.4°. Smaller, broad peaks at 50,4°, 54.0° and 77.2° can also be correlated to the MWCNT structure. The broadened peaks are typical for diffraction patterns of NPs, implying a small crystallite size.^38,42^ The XRD diffraction patterns from 30° to 60° of Fe-MWCNTs, Fe_3_O_4_-MWCNTs, Co-MWCNTs, and Ni-MWCNTs are presented in Figure 11. This 2theta (2θ) range was chosen to disregard the strong graphite peak at 26.2° and since this region had the most distinct features for each sample. All peaks appearing in the MWCNT diffraction pattern were also present in the XRD patterns of all other samples. These peaks included signal scattering from the MWCNT structure at 42.2°, 44.4°, 50.4°, and 54.0°, as well as from Fe catalyst NPs at 43.7°. It should be noted that two additional peaks around 40.0° and 48.7° were detected in all the patterns. These peaks were not identified but are assumed to correspond to MWCNTs or Fe catalyst NPs. The Fe-MWCNT diffraction pattern was matched with the reference patterns of graphite (COD 1 200 017), pure Fe (COD 9 016 291), and Fe_3_O_4_ (COD 1 532 796). Reference patterns of α-Fe_2_O_3_ (COD 1 011 240) and maghemite (γ- Fe_2_O_3_) (COD 9 006 316) were also included for comparison. The broad peak with a local maximum at 35.7° overlapped with the main Fe_3_O_4_ peak at 35.5°, one of the α-Fe_2_O_3_ peaks at 35.6° and the γ-Fe_2_O_3_ peak at 35.7°. To determine whether the Fe-MWCNT pattern matched the Fe_3_O_4_, α-Fe_2_O_3_ or γ-Fe_2_O_3_ patterns better, more peaks were compared. The peak at 37.3° could be matched with the Fe_3_O_4_ peak at 37.1° or the γ- Fe_2_O_3_ peak at 37.4°. In addition, the broad peak (40.1° – 46.3°) showed a longer, left tail compared to the Fe_3_O_4_-MWCNT, Co-MWCNT, and Ni-MWCNTs patterns, which the Fe peak at 43.7°, the Fe_3_O_4_ peaks at 40.2°, 43.1° and 44.5°, the α-Fe_2_O_3_ peak at 43.5° or the γ-Fe_2_O_3_ peak at 43.4° could be responsible for. The broad peak centered at 53.6° originated from signal scattering of the MWCNT structure at 54.0°, and the Fe_3_O_4_ peak at 53.5°, the α-Fe_2_O_3_ peak at 54.0° or the γ-Fe_2_O_3_ peak at 53.9°. With that, many characteristic peaks of the Fe_3_O_4_ and γ-Fe_2_O_3_ patterns matched the one of the Fe-MWCNTs. Contrarily, the main α-Fe_2_O_3_ peak at 33.1°, as well as another peak at 49.4° were not present in the Fe-MWCNT pattern. This does not rule out the possibility that no α-Fe_2_O_3_ was contained in the Fe-based NP oxide layer. However, the dominant iron oxide structure was Fe_3_O_4_ or γ-Fe_2_O_3_ according to XRD analysis results. A protective iron oxide layer is advantageous as it enhances the stability and biocompatibility of the NPs, compared to pure Fe NPs.^43,44^ The match between the Fe_3_O_4_-MWCNT diffraction pattern and the Fe_3_O_4_ or γ-Fe_2_O_3_ reference patterns was more obvious, particularly with the main peak at 35.4°. All the main Fe_3_O_4_ and γ-Fe_2_O_3_ peaks were identifiable in the Fe_3_O_4_-MWCNT pattern in addition to the MWCNT and Fe catalyst NP signals. The peak at 43.1° within the broad peak (42.0° – 46.2°) grew significantly taller compared to the other spectra due to the strong Fe_3_O_4_ or γ-Fe_2_O_3_ signal. Again, several characteristic α-Fe_2_O_3_ peaks were not detected. As such, it can be deduced that Fe_3_O_4_ or γ-Fe_2_O_3_, instead of α-Fe_2_O_3_ were the dominant iron oxide phases as per XRD results. The distinction between Fe_3_O_4_ or γ-Fe_2_O_3_ phases was more evident in the SAED and XPS analyses. The Co-MWCNT diffraction pattern was matched with Co (COD 9 012 884) and CoO (COD 9 008 618) reference patterns. The main Co and CoO peaks at 43.6° and 42.3°, respectively, overlapped with the two highest peaks within the broad peak (41.4° – 46.2°). It needs to be noted that there was overlap between the 42.3 °CoO peak and the 42.2° MWCNT peak, as well as the 43.6 °Co peak and the 43.7 °Fe peak. As such, these components may have contributed to the intensity of those peaks. The second CoO peak also matched the peak at 37.3°. The remaining Co peaks fit the experimental diffraction patterns well for peaks above 35°. It is possible that due to the small mass of Co in the Co-MWCNT sample, only the strongest Co peaks were distinguishable from signal noise. Finally, the Ni-MWCNT diffraction pattern was compared to Ni (COD 4 320 504) and NiO (COD 9 008 693) reference patterns. Similar to Co-MWCNTs, the main Ni and NiO peaks at 44.3° and 43.4°, respectively, were within the range of the broad peak (41.4° – 45.9°). The prominent peak at 42.4° is attributable to the graphitic structure of the MWCNTs. Additionally, overlap of the NiO peak with the peak at 37.3° was observed. A small Ni peak at 51.6° was also detected. Due to the presence of comparingly few NPs on the samples and overlap between reference patterns (Fe_3_O_4_ and γ- Fe_2_O_3_), XRD analysis of the m-MWCNTs proved to be difficult. However, the comparison with reference diffraction patterns of bulk materials gave further insight into the crystallographic structure of the deposited NPs. It should be kept in mind that small discrepancies in 2θ values of the measured peaks versus the reference values may be due to the NP morphology and shape.^45^

**Figure 12:**
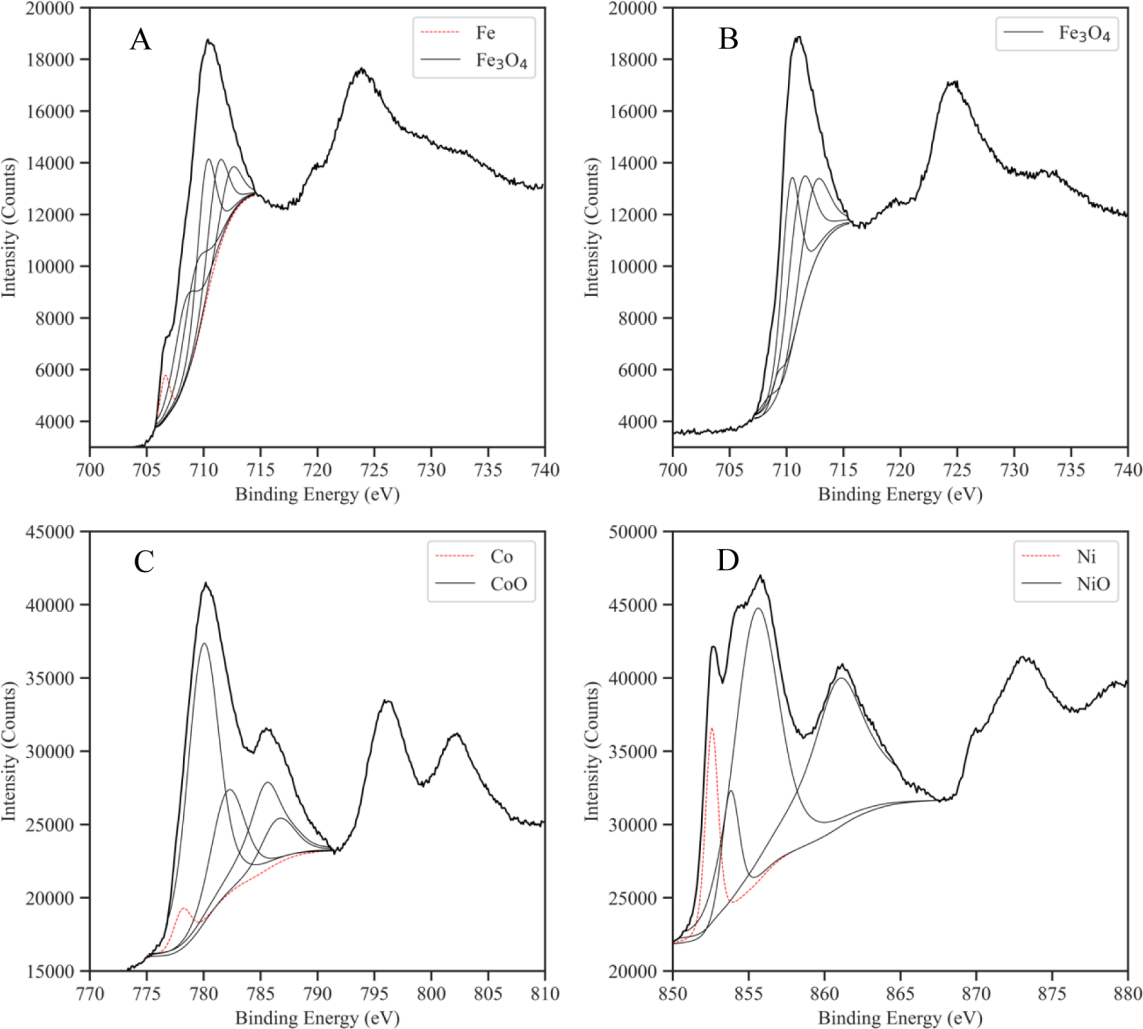
XPS high-resolution spectra of (A) Fe-MWCNTs, (B) Fe_3_O_4_-MWCNTs, (C) Co-MWCNTs, and (D) Ni-MWCNTs. For Fe-MWCNTs, Co-MWCNTs, and Ni-MWCNTs, the spectra are fit with characteristic peaks of the pure target metal and its respective oxides. For Fe_3_O_4_-MWCNTs, characteristic Fe_3_O_4_ peaks are used for spectrum fitting.

To better understand the elemental composition of the deposited NPs, high-resolution XPS and peak deconvolution were performed as shown in Figure 12. For each sample, a peak representing a metallic element is illustrated as a dotted red line. The thin solid black lines denote metallic oxides. The main difference between the Fe-MWCNT and Fe_3_O_4_-MWCNT spectra is the shoulder on the first peak in the Fe-MWCNT spectrum. For the Fe-MWCNTs, the high-resolution Fe spectrum was fitted with peaks centered at binding energies of 706.6 (Fe), 708.4 (Fe_3_O_4_), 709.2 (Fe_3_O_4_), 710.2 (Fe_3_O_4_), 711.5 (Fe_3_O_4_), and 712.3 eV (Fe_3_O_4_).^46^ These results confirm the assumption of a thin Fe_3_O_4_ shell covering an Fe core. For the Fe_3_O_4_-MWCNTs, the high-resolution Fe spectrum was fitted with peaks centered at 708.3 (Fe_3_O_4_), 709.2 (Fe_3_O_4_), 710.2 (Fe_3_O_4_), 711.7 (Fe_3_O_4_), and 712.3 eV (Fe_3_O_4_).^46^ The Co-MWCNTs contained elemental Co (778.1 eV), as well as CoO. The corresponding high-resolution spectrum was fitted with characteristic CoO peaks at 780.0, 782.2, 785.4, and 786.5 eV.^47^ A large elemental Ni peak appeared in the high-resolution Ni spectrum of the Ni-MWCNT sample which was centered at a binding energy of 852.6 eV. NiO peaks were centered at 853.7, 855.5, and 860.9 eV.^48^

**Figure 13:**
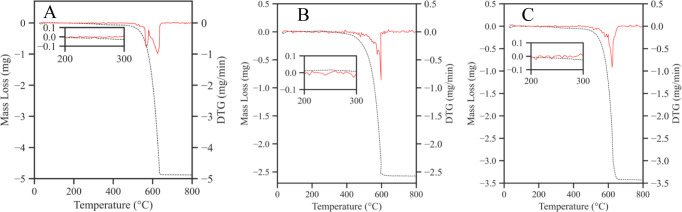
TGA mass loss graphs of (A) MWCNTs, (B) Fe-MWCNTs, and (C) Fe_3_O_4_-MWCNTs. A small mass increase for the Fe-MWCNTs and Fe_3_O_4_-MWCNTs samples between 200 and 300 °C denotes the oxidation of Fe_3_O_4_ to α-Fe_2_O_3_.

The composition of the NPs from the Fe and Fe_3_O_4_ targets was further investigated by TGA in air. Pristine MWCNTs without NPs were also tested as a baseline. The mass loss and the derivative thermogravimetric curves for each sample are displayed in Figure 13. No significant mass loss was observed up to a temperature of 100 °C, indicating that no water was adsorbed on any of the sample surfaces. A slight mass increase was measured for the Fe-MWCNT and Fe_3_O_4_-MWCNT samples at temperatures between 200 and 300 °C. The average mass increase in this temperature range was 0.38 ± 0.09% and 0.33 ± 0.01% for the Fe-MWCNT and Fe_3_O_4_-MWCNT samples, respectively. On the contrary, the mass of pristine MWCNTs decreased by 0.06 ± 0.04% on average in the same temperature range. In the 200–300 °C temperature range, the most important transition taking place is attributable to the oxidation of Fe_3_O_4_ to α-Fe_2_O_3_ according to the reaction below:^42,49^
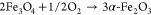


A possible explanation as to why a higher mass increase was observed with the Fe-MWCNT as opposed to the Fe_3_O_4_-MWCNT sample could be the oxidation of pure Fe to α-Fe_2_O_3_. This reaction can happen with or without intermediate reactions:
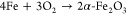


It needs to be remembered that most of the samples’ mass came from MWCNTs as opposed to the Fe-based NPs which underwent oxidation. This explains the small mass differences and the importance of having a MWCNT reference sample. Eventually, the complete oxidation of MWCNTs into gaseous CO and CO_2_ took place:^42^
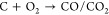


MWCNT oxidation took place in the 515–665 °C temperature range which is in agreement with values reported previously.^50^ The temperature at which the MWCNTs were fully oxidized was the same across all tested samples. However, the onset temperature was lower for the Fe-MWCNTs and Fe_3_O_4_-MWCNTs due to the catalytic activity of Fe-based NPs for MWCNT oxidation.^38^ After TGA, α-Fe_2_O_3_ (rust) was the only residue left in the sample holder. For MWCNTs, the remaining mass came to 5.23 ± 3.15% of the total mass. The source of the remaining α-Fe_2_O_3_ of the pristine MWCNTs were the Fe catalyst particles located inside individual MWCNTs.^36^ The remaining mass of Fe-MWCNTs and Fe_3_O_4_-MWCNTs were 16.57 ± 3.46% and 16.39 ± 0.96%, respectively. Of course, the higher remaining sample masses were due to the Fe-based NPs which were deposited during PLD.

**Figure 14:**
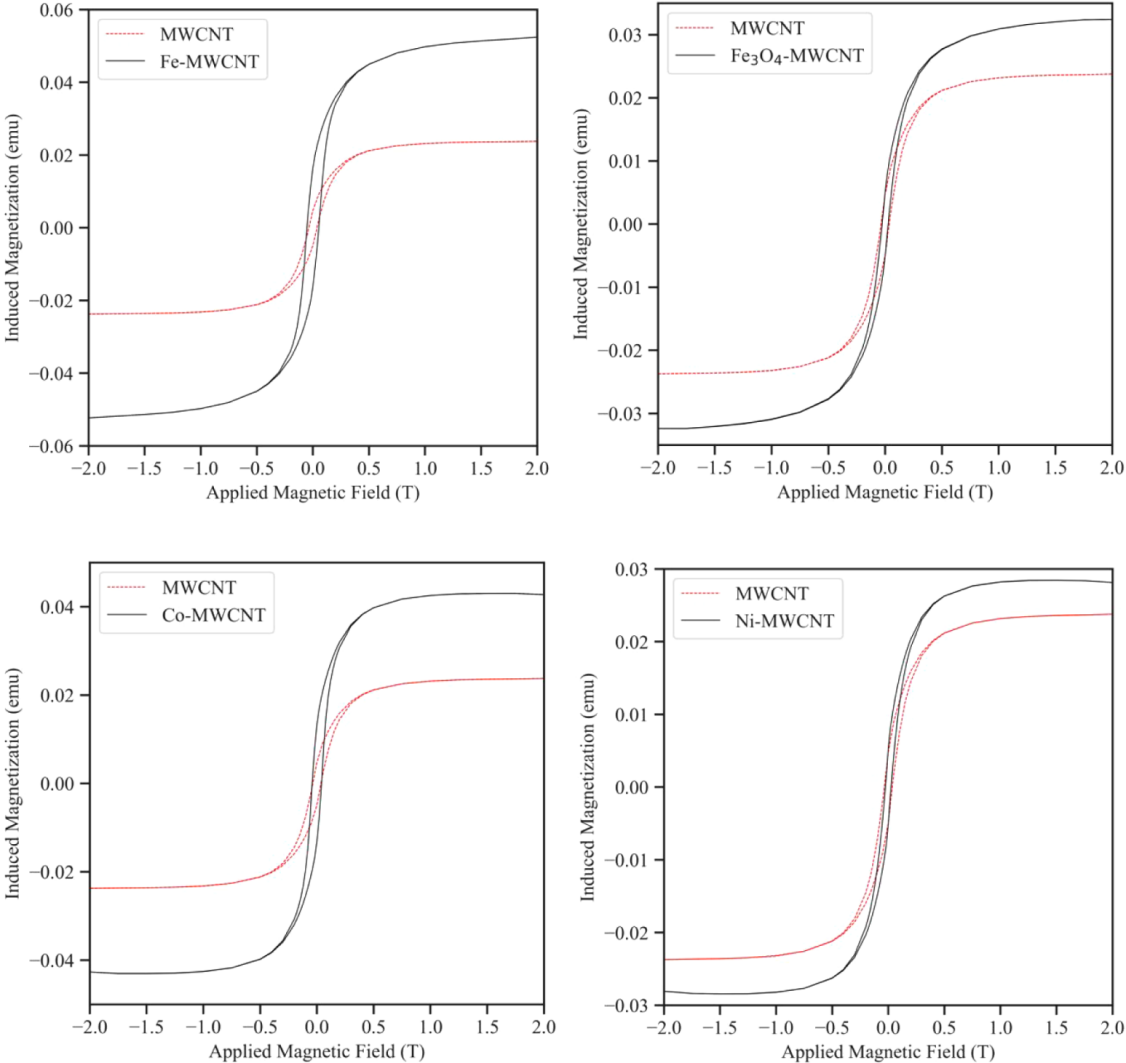
M vs H curves of Fe-MWCNTs, Fe_3_O_4_-MWCNTs, Co-MWCNTs, and Ni-MWCNTs in comparison with MWCNTs. For all samples, the maximum induced magnetization increases compared to pristine MWCNTs.

The magnetic properties of pristine and m-MWCNTs were evaluated by VSM. Figure 14 shows the characteristic M vs H curves for Fe-MWCNT, Fe_3_O_4_-MWCNT, Co-MWCNT, and Ni-MWCNT samples in comparison with pristine MWCNTs. A multitude of parameters characteristic of the sample being tested can be read from an H vs M curve. The magnetic saturation (M_s_) marks the maximum induced magnetization. Once M_s_ is reached, the induced magnetization will not increase further regardless of the magnitude of the applied magnetic field (H). The remanence or remanent magnetization (M_r_) measures the remaining magnetization in the tested sample when a large magnetic field has returned to zero after fully magnetizing the material. Closely related is the coercivity or coercive force (H_c_) which is the magnetic field strength required to fully demagnetize the material. Lastly, the magnetic susceptibility (χ_m_), which is given by the slope of the H vs M curve, quantitively describes the magnetization response of the sample to the applied field.^16,51^ The main findings are summarized in Table 3.

**Table 3:** Magnetic Property Data of MWCNTs, Fe-MWCNTs, Fe_3_O_4_-MWCNTs, Co-MWCNTs, and Ni-MWCNTs

Sample	H_c_ [T]	M_r_ [emu/g]	Average M_s_ [emu/g]	Max. induced magnetization by NP addition [emu/g]	Ms of bulk material [emu/g]
MWCNT	0.030 ± 0.002	0.004 ± 0.001	2.46 ± 0.08	/	/
Fe-MWCNT	0.042 ± 0.015	0.014 ± 0.004	5.37 ± 0.15	2.91	217.9
Fe_3_O_4_-MWCNT	0.026 ± 0.003	0.007 ± 0.002	3.79 ± 0.38	1.33	90–92
Co-MWCNT	0.032 ± 0.011	0.008 ± 0.004	4.29 ± 0.01	1.83	162.7
Ni-MWCNT	0.030 ± 0.007	0.005 ± 0.001	2.85 ± 0.06	0.40	57.5

As mentioned previously, Fe catalyst NPs are dislodged from the SS growth surface and entrapped inside individual MWCNTs during chemical vapor deposition. Therefore, the as-synthesized (pristine) MWCNTs exhibit magnetic properties and reached a M_s_ value of 2.45 emu/g for an applied magnetic field of 2 T. The M_s_ values were 5.37 emu/g, 3.79 emu/g, 4.29 emu/g, and 2.85 emu/g for Fe-MWCNTs, Fe_3_O_4_-MWCNTs, Co-MWCNTs, and Ni-MWCNTs, respectively. The added magnetic content was quantified using the ratio M_S_/M_So_, where M_s_ is the magnetic saturation of m-MWCNT samples and M_So_ is the magnetic saturation of pristine MWCNTs.^49^ The added magnetic content was 219% for Fe-MWCNT, 175% for Co-MWCNT, 155% for Fe_3_O_4_-MWCNT, and 116% for Ni-MWCNT samples. While PLD using Fe, Fe_3_O_4_, and Co targets increased the M_s_ compared to pristine MWCNTs by at least more than half, the deposition of Ni-based NPs did not amplify the magnetic properties of MWCNTs much. The trend of how much NPs from each target increased the M_s_ in comparison with pristine MWCNTs matched that of the bulk material M_s_ values (cf. Table 3). The addition of NPs from the Fe target led to the highest M_s_ because the Fe atom has a stronger magnetic moment compared to Co or Ni which is due to the presence of four unpaired electrons in the atom’s 3d orbital in a pure Fe configuration. This changes as Fe atoms form oxides, thus explaining the reduced magnetization effect associated with the addition of Fe_3_O_4_ NPs.^49^

The M vs H curves of the pristine and m-MWCNTs had similar appearances with narrow hysteresis loops. These hysteresis loops represent magnetic energy losses. Typically, magnetic NPs can achieve magnetic saturation in the presence of a magnetic field without displaying magnetic hysteresis. This is because sufficient thermal energy is supplied, even at room temperature, to overcome the energy barrier for the magnetic moment of the single domain NP to change. This phenomenon is described as superparamagnetic relaxation and is characteristic of magnetic NPs.^44^ The NPs of the m-MWCNTs were composed of single magnetic domains as they were on the order of a few nm in size. Since the magnetic susceptibility increased for m-MWCNTs compared to pristine MWCNTs, superparamagnetic behavior of the NPs can be assumed.^44,49^ The small hysteresis loops and nonzero coercive field behavior of the m-MWCNTs may originate from the iron catalyst particles within the MWCNTs as they were already detected for pristine MWCNT samples. The *τ*_*M*_ of the VSM was on the order of 0.1 s which largely exceeds the *τ*_*N*_ of the deposited NPs at room temperature. This suggests that they were in a superparamagnetic state. Due to the application of an external field during VSM measurements at room temperature, the magnetic spins of the NPs aligned with the field lines which allowed for the detection of induced magnetization values for the range of tested field strengths.^52^ It can further be seen that all tested samples reached magnetic saturation well within the tested field range. Static or low-frequency and low-magnetic fields do not interact with tissues and organs. The maximum tested field strength (*H*_max_) of ±2 T and the measuring frequency (f_m_) of 75 Hz were selected such that *H*_max_·f_m_ < 5 × 10^8^ A/(ms). This value marks the onset above which damage to living tissue could occur.^53,54^ Therefore, the presented results could be considered for biomedical applications.

Lastly, the adhesion of the deposited NPs to the MWCNTs was evaluated. ICP-OES results (cf. Table 4) revealed the absence of Fe in testing solutions obtained with Fe-MWCNTs and Fe_3_O_4_-MWCNTs. We hypothesize that the NPs exhibiting coarse or film-like deposition patterns partially solidified on the MWCNTs and disrupted the underlying graphitic structure. As a result, they formed strong bonds with the MWCNT walls. On the contrary, approximately 10 μg Co and Ni were measured in the testing solutions of Co-MWCNTs and Ni-MWCNTs, respectively. For Co-MWCNTs, this marked a NP mass loss of 3.75% and for Ni-MWCNTs, a NP mass loss of 4.22% after sonication for an hour. These results indicate that the Co and Ni-based NPs remained on individual MWCNTs partially by weak adhesion, possibly because Co and Ni-based NPs reached the MWCNTs as solidified particles.

**Table 4:** ICP-OES Results of Fe-MWCNTs, Fe_3_O_4_-MWCNTs, Co-MWCNTs, and Ni-MWCNTs

Sample	Mass of Fe detected in solution [μg]	Mass of Co detected in solution [μg]	Mass of Ni detected in solution [μg]	Total NP mass loss [%]
Fe-MWCNT	nil	/	/	0.00
Fe_3_O_4_-MWCNT	nil	/	/	0.00
Co-MWCNT	/	10.50	/	3.75
Ni-MWCNT	/	/	9.29	4.22

## 4 Conclusion

Magnetic MWCNTs were prepared by NP addition using dry PLD with Fe, Fe_3_O_4_, Co, and Ni targets. The morphology and total mass of the deposited NPs varied based on the process parameters and physical properties of the target. When characterizing the magnetic NPs, it is crucial to consider the results of all the performed characterization techniques and to ensure that the combination of these leads to the same conclusions. This was the case in the present investigation. Most of the deposited NPs were composed of the pure target metals and their oxides: Fe@Fe_3_O_4_ NPs for the Fe target, Co@CoO NPs for the Co target, and Ni@NiO NPs for the Ni target. The only exception are the NPs from the Fe_3_O_4_ target which produced pure Fe_3_O_4_ NPs. The Fe@Fe_3_O_4_ NPs from the Fe target were most successful at increasing the magnetization of MWCNTs, while the NPs originating from all the other targets contributed less. The superior adhesion of Fe@Fe_3_O_4_ and Fe_3_O_4_ NPs to MWCNTs compared to Co@CoO and Ni@NiO NPs favors the use of Fe and Fe_3_O_4_ targets for the production of stable m-MWCNTs. As such, the final application of the proposed functional materials should not be chosen solely based on their magnetic properties but should encompass their entire set of properties.

## Abbreviations

Fe: iron
Fe_3_O_4_: magnetite
α-Fe_2_O_3_: hematite
γ-Fe_2_O_3_: maghemite
Co: cobalt
Ni: nickel
Fe@Fe_3_O_4_: Fe nanoparticle with a Fe_3_O_4_ shell
Co@CoO: Co nanoparticle with a CoO shell
Ni@NiO: Ni nanoparticle with a NiO shell
CNT: carbon nanotube
MWCNT: multiwalled carbon nanotube
NP: nanoparticle
PLD: pulsed laser deposition
m-MWCNT: magnetic nanoparticle-covered multiwalled carbon nanotube
TEM: transmission electron microscopy
EDS: energy-dispersive X-ray spectroscopy
SAED: selected area electron diffraction
XRD: X-ray diffraction
XPS: X-ray photoelectron spectroscopy
TGA-TPO: thermogravimetric analysis with temperature-programmed oxidation
VSM: vibrating sample magnetometer
ICP-OES: inductively coupled plasma optical emission spectroscopy
SS: stainless steel
Fe-MWCNTs: multiwalled carbon nanotubes with iron-based nanoparticles
Fe_3_O_4_-MWCNTs: multiwalled carbon nanotubes with magnetite-based nanoparticles
Co-MWCNTs: multiwalled carbon nanotubes with cobalt-based nanoparticles
Ni-MWCNTs: multiwalled carbon nanotubes with nickel-based nanoparticles
OES: optical emission spectroscopy
COD: Crystallography Open Database
